# Believing Is Seeing: Fixation Duration Predicts Implicit Negative Attitudes

**DOI:** 10.1371/journal.pone.0105106

**Published:** 2014-08-18

**Authors:** Maria Laura Mele, Stefano Federici, John Lawrence Dennis

**Affiliations:** 1 Department of Philosophy, Social & Human Sciences and Education, Perugia, Italy; 2 ECONA, Interuniversity Centre for Research on Cognitive Processing in Natural and Artificial Systems, Sapienza University of Rome, Rome, Italy; 3 Department of Psychology, Catholic University, Milan, Italy; 4 The Umbra Institute, Perugia, Italy; Barrow Neurological Institute, United States of America

## Abstract

A prototypical finding of social cognition is that social experiences influence later performance even though those experiences are not introspectively available. Building on social cognition research on implicit attitudes, we evaluate whether ethnic category/attribute pairs influence eye movements during the Implicit Association Test (IAT, Greenwald, McGhee, & Schwartz 1998). Results show that fixation duration predicted implicit attitudes such that when the category/attribute pairs disconfirmed one's implicit negative attitude fixation duration toward that pair increased. The present research provides evidence that eye movements and implicit processes inherent in the IAT are more broadly connected than previously thought.

## Introduction

Understanding people's beliefs, feelings, and attitudes is often difficult. Reasons for these difficulties vary greatly, but social desirability [1], [2] and the inaccessibility of psychological processes [3] are two of the most commonly cited reasons. To overcome these difficulties, implicit techniques, like the Implicit Association Test (IAT) have been developed [4].

The classic IAT reveals implicit attitudes towards ethnic groups by asking people to associate one of two ethnic categories (e.g., white vs. black) with a bipolar attribute (e.g., good vs. bad). When the category/attribute pairs are highly associated, response accuracy increases and association time associate decreases. Because of its flexibility, robustness, and reliability the IAT has been widely used to study automatic processes [5].

Previous research has demonstrated that indirect behavioral measurement methods, like those found in the IAT, e.g., response times, and eye-tracking techniques are good indicators of implicit processes [6], [7]. Interestingly, the relationship between these two indirect behavioral measurement methods has only recently been studied [8]–[10], and it is still unclear whether eye movements can provide a predictive model of implicit processes. The present research attempts to do just that.

The present research is understood within an embodied cognition theoretical framework. Embodied cognition has repeatedly demonstrated that bodily experiences work in concert with cognitive systems underlying sensory perception, action, emotion, motivation, and cognitive operations [11], [12]. Research has found that social information processing can occur via bottom-up processes such that bodily experiences influence high level cognitive processes or via top-down processes such that cognition directly influences sensory-motor processes [11], [13], [14]. We hypothesize a top-down oculo-sensory-motor embodiment of social information processing, in line with a growing number of studies on the perception of social stimuli being associated with bodily states [14]–[16].

Visual attention is guided towards unexpected content [17], [18] that taxes cognitive load [19]. In fact, considering this relationship between visual attention and cognitive load, participants should show more and/or longer fixations towards visual areas that disconfirm one's implicit negative attitude towards the ethnic out-group, i.e., black/good consistent with research on the salience of negative attitudes towards out-groups [20], [21]. Eye movements should therefore integrate with belief systems that underlie implicit attitudes [14], [22], [23]. Such a finding would allow eye movements to be considered as a predictive tool for psychological concepts like attitudes [24].

The present research investigated the relationship between eye movements and the IAT, an excellent measure of implicit processing. Eye movements are increasingly being used in different fields (see e.g., the neuroergonomic studies conducted by Di Stasi and colleagues [25]) as a bio-behavioral measure for different physiological and psychological states, such as arousal [26], and cognitive and attentional load [25]–[29]. By using an “off the shelf” eye-tracking methodology with a traditional Black-White IAT, two studies were conducted. Study 1a established that there was a relationship between eye movements and the IAT while Study 1b refined the methodology. Together, these two studies suggest that fixation duration increased when the visual stimuli disconfirmed the implicit negative attitude towards the ethnic out-group, i.e., black/good. Fixation duration, therefore, was found to predict implicit attitudes towards the ethnic out-group.

## Study 1a

Participants were presented with the IAT while fixation number and duration were measured. Considering the previous discussion in the Introduction on the relationship between attention and unexpected visual information, a positive relationship between fixation number and/or duration and implicit attitudes, as measured by the IAT, was expected.

### Method

#### Materials

Eye movements were measured using the ITU Gaze Tracker software (www.gazegroup.org) that records gaze position via a webcam that reflects infrared light on the cornea. Following a nine-point calibration, the ITU device tracks eye movements with a mean error in visual angle degrees of 1.48 (SD = 0.58) [30].

A Milliseconds Racism IAT (http://www.millisecond.com/download/library/IAT) combining the two Caucasian and African ethnic categories with the good or bad qualitative attributes was administered online, whereas the OGAMA IAT was based on the online IAT structure using OGAMA's Slideshow Design Module. The IAT was administered twice (the IAT can be administered more than once with one having little or no impact on the others [31]). The online IAT was used to calculate implicit association score whereas the OGAMA IAT was used to measure and analyze the eye movements performed during the visual interaction with the IAT stimuli.

#### Participants

30 Caucasian (15 female; age *M* = 34; *SD* = 4.31, 80% right-handed; all with 100% visual acuity, 33% with contact lenses) randomly completed either the online IAT or the OGAMA IAT first. The study was reviewed and approved by the Institutional Review Board of the Department of Philosophy, Social & Human Sciences and Education, University of Perugia. All participants provided their written informed consent to participate in this study. No minors/children were enrolled in this study. The study represented “no more than minimal risk”.

#### Design and Procedure

Sessions were conducted in a quiet setting and they began with a visual acuity and eye dominance assessment. Participants were asked to complete either the online IAT or the OGAMA IAT first. For both participants, semantically associated words or pictures shown in the middle of the screen to their corresponding category shown either on the left or the right via keyboard presses.

Monocular eye movements were sampled by the ITU Gaze Tracker through an infrared cam mounted on an adjustable chinrest support that was positioned 60 cm from the screen. Only during the OGAMA IAT were eye movement fixation number and duration measured. Fixations were calculated using the dispersion-type detection algorithm by LC technologies [32]. We set the maximum distance that a point may vary from the average fixation point at 20 pixels and the minimum number of samples that define a fixation at 5 samples. Consecutive fixations within the maximum distance were merged into one fixation. Two Areas Of Interest (AOIs) have been defined on a 1024×768 LCD monitor: a rectangular Left AOI, coordinates = P0:(−0.7;0.0) P1:(646.3;0.0) P2:(646.3;268.8) P3:(−0.7;268.8), and a Right AOI, coordinates = P0:(644.5;−1.9) P1:(1282.7;−1.9) P2:(1282.7;268.8) P3:(644.5;268.8).

Participants were asked to associate one of two ethnic categories (e.g., white vs. black) with a bipolar attribute (e.g., good vs. bad), both presented on a screen. The OGAMA IAT consisted of three blocks: one control and two experimental (i.e., initial and reversed). Each block contained nineteen trials where the screen position of the ethnic categories (black/white) varied between blocks while the attributes (bad/good) were fixed for all trails. The duration of those trials depended on the amount of time participants' took to respond to the IAT via key presses. In the control blocks, the ethnic categories of black and white were presented either on the left or right while the qualitative attribute good was always presented on the left and bad was always presented on the right. Therefore, for the initial blocks, the category/attribute pair white/good was presented on the left and black/bad was presented on the right while for the reversed blocks, black/good was presented on the left and white/bad was presented on the right (see Table 1 for a category/attribute pair schema).

**Table 1:** Categories, attributes and category/attribute pairs and their positions for the Black-White Implicit Association Test (IAT) used in Study 1b.

Condition	Control	Control	Initial blocks	Reversed blocks
Good Left	•White	•Good	•White/Good	•Black/Good
	Black•	Bad•	Black/Bad•	White/Bad•
Good Right	•White	Good•	•White/Bad	•Black/Bad
	Black•	•Bad	Black/Good•	White/Good•

The black dots on the table indicate the left or right position of the target on the screen. Good Left corresponds to what was presented in Study 1a.

### Results

IAT results revealed that 86% of the participants showed an automatic preference for white people (33% strong, 33% moderate and 20% slight preference), whereas 7% showed a slight automatic preference for black people and 7% had no automatic preference.

A repeated-measures ANOVA on fixation number demonstrated a main effect of condition (*F*(2,28) = 4.198, *p* = .025) and a main effect of position (left, right) (*F*(1,29) = 4.677, *p* = .039). No significant interaction was found between condition and position (*F*(2,28) = 1.033, *p*>.05). A repeated-measures ANOVA on fixation duration demonstrated no main effect of condition and position. No significant interaction was found between condition and position too.

Fixation analysis on category/attribute pair combination within each experimental block revealed that, for those with an automatic white people preference, in initial blocks, fixation number for the pair black/bad (*M* = 2.93; *SD* = 5.38 fixation count per AOI) was significantly lower than white/good (*M* = 7.8; *SD* = 14.8 fixation count per AOI) *F*(1, 29) = 14.34, *p*<.05, while for the reversed blocks no difference between the black/good (*M* = 4.5; *SD* = 6.4 fixation count per AOI) and white/bad pairs was found (*M* = 3.73; *SD* = 7.4 fixation count per AOI) *F*(1, 29) = .237, *p*>.05. Fixation duration was higher for black/bad (*M* = 1374.3; *SD* = 2586.5 ms) than white/good (*M* = 810.7; *SD* = 2556.3 ms) in the initial blocks *F*(1, 29) = 7.85, *p*<.05 and black/good (*M* = 1442.5; *SD* = 2649.1 ms) than white/bad (*M* = 1212.5; *SD* = 2791.2 ms) in the reversed blocks *F*(1, 29) = 3.38, *p*<.05.

Multiple linear regression analysis showed that fixation number and duration were not able to predict automatic preferences *R^2^* = .029, *F*(2, 27) = .415, *p*>.05, fixation number β = .118, p> .05; fixation duration *β* = .064, *p*>.05, although the intercept suggested a trend effect (Intercept = .85; *t*(27) = 8.09; *p*<.01). A significant correlation between fixation number and fixation duration was found for both initial (*r* = 0.95, *p*<.05) and reversed (*r* = 0.52, *p*<.05) blocks.

### Discussion

**Figure 1:**
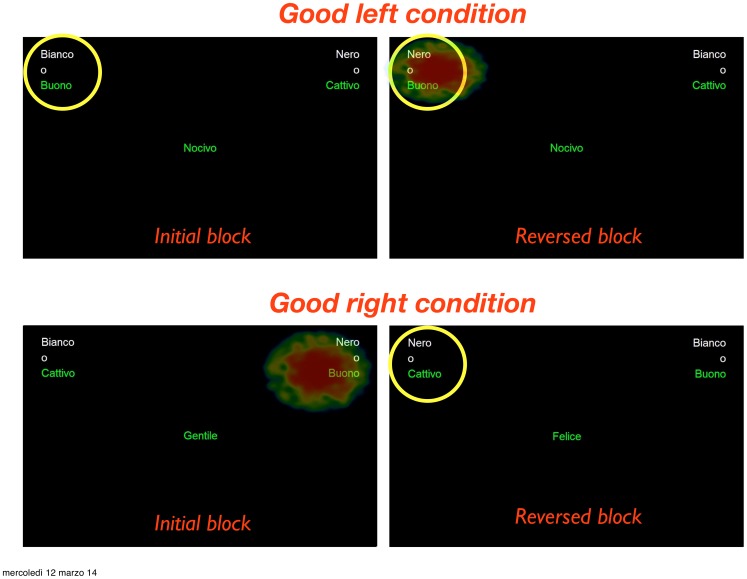
The screenshots show the 2×2×2 combination of the ethnic category “nero” (black) and “bianco” (white) with the qualitative attributes “buono” (good) and “cattivo” (bad) for both good left and good right conditions. For each experimental condition, a yellow circle represents the effect of position on fixation number whereas the heat map represents the effect of category-attribute combination on fixation duration.

Results suggest that fixation number was significantly different among both condition and position, although condition did not influence gaze position on AOIs. Fixation duration did not differ between blocks. A trend effect for the relationship between fixation number/duration and implicit attitudes, as measured by the IAT, was found. Participants fixated more and longer on black/bad than white/good while also fixating longer on black/good than white/bad. Results demonstrate that category/attribute pairs that confirm as well as disconfirm one's implicit attitudes toward an out-group ethnic category are those that attract visual attention. Since the visual targets used for each AOI (i.e., the pairs of words described in fig. 1) never vary in color, contrast, texture, line shape, size, orientation and background during the trials, we exclude the possibility that fixation duration differences would primarily be influenced by visual stimuli, as recently highlighted by McCamy and colleagues [33]. These results demonstrate that eye tracking is a good candidate for indirectly measuring implicit processes [6]–[10].

Even though automatic preferences are not influenced by lateralization [4], eye movements can be due to an upper-left gaze bias [34]. In this study the attributes good/bad were always presented in fixed positions. Therefore, white/good and black/good were only presented on the left while white/bad and black/bad were only presented on the right. These fixed positions could have led to an eye movement lateralization effect. Since most people read from the upper left to the lower right [35] it is difficult to conclude whether one's gaze toward the pair was due to the pair's salience or reading lateralization. For this reason, the methodological design behind the IAT cannot exclude or explain any influence of lateralization on strategies used to explore one's visual space. Study 1b was conducted to resolve this problem.

## Study 1b

Identical to Study 1a participants completed the Black-White IAT while fixation number and duration were measured. In Study 1b a between subjects 2×2×2 experimental design (category x attribute x position) was used to control for a possible eye-movement lateralization effect. Considering the results from Study 1a, we predicted that category/attribute pairs that confirm/disconfirm one's implicit attitudes toward an out-group ethnic category are those that attract visual attention.

### Method

#### Materials

Identical to Study 1, ITU Gaze Tracking software, the online IAT and the OGAMA IAT were used.

#### Participants

48 Caucasians (29 female; *M* = 23.5; *SD* = 7.35; 85.4% right handed; all with 100% visual acuity, 20% with contact lenses) were randomly assigned to perform either the online IAT or the OGAMA IAT first. Identical to Study 1, the experiment was reviewed and approved by the Institutional Review Board of the Department of Philosophy, Social & Human Sciences and Education, University of Perugia. All participants provided their written informed consent to participate in this study.

#### Design and Procedure

The design and procedure for this study were identical to Study 1a except that in the initial and reversed blocks for Study 1b, position on the screen (left/right) of two ethnic categories (black/white) and qualitative attributes (good/bad) was manipulated in a 2×2×2 between subjects design. Two experimental conditions: good left and good right, where the positive attribute good was fixed on either the left or right were administered. Good left corresponded to what was presented in Study 1. See Table 1 for a representation of the category/attribute pairs.

### Results

Results from the online IAT revealed that 67% of participants showed an automatic preference for white people (*N* = 32, of which 33% strong, 7% moderate and 60% slight preference), while 33% (*N* = 16) of the participants had no automatic preference. No significant difference between the two groups was found *F*(1, 45) = 0.49, *p*>.05.

For the good left condition, a repeated-measures ANOVA on fixation number demonstrated a main effect of condition (*F*(2,23) = 10.427, *p* = .001) and a main effect of position (left, right) (*F*(1,23) = 9.120, *p* = .006). A significant interaction was found between condition and position (*F*(2,23) = 5.202, *p* = .014). A repeated-measures ANOVA on fixation duration demonstrated a main effect of condition (*F*(2,23) = 6.211, *p* = .007) and position (left, right) (*F*(1,24) = 7.096, *p* = .014). No significant interaction was found between condition and position.

For the good right condition, a repeated-measures ANOVA on fixation number demonstrated a main effect of condition (*F*(2,20) = 6.464, *p* = .007). No significant differences were found for position (left, right) (*F*(1,21) = 0.041, *p*>.05). No significant interaction was found between condition and position (*F*(2,20) = 2.388, *p*>.05). A repeated-measures ANOVA on fixation duration demonstrated a main effect of condition (*F*(2,20) = 8.699, *p* = .002) and no effect of position. No significant interaction was found between condition and position, although we found a trend of interaction (*F*(2,20) = 2.936, 0<*p<1*).

Fixation analysis revealed that for those with an automatic white people preference, only fixation number was influenced by the left target position for all block/condition pairs, *Wilks lambda* = .76, *F*(4, 39) = 3.14, *p*<.05. In the good left condition for the initial blocks fixation number for the pair white/good (*M* = 22.9; *SD* = 31.4 fixation count per AOI) was significantly higher than black/bad (*M* = 8.7; *SD* = 14.1 fixation count per AOI); *F*(1, 24) = 4.24, *p*<.05 while for the reversed blocks, black/good (*M* = 10.2; *SD* = 20.5 fixation count per AOI) was significantly higher than white/bad (*M* = .72; *SD* = 1.54 fixation count per AOI); *F*(1, 24) = 5.26, *p*<.05. In the good right condition reversed blocks, fixation number for the pair black/bad (*M* = 14.7; *SD* = 24 fixation count per AOI) was significantly higher than white/good (*M* = 4.7; *SD* = 6.4 fixation count per AOI); *F*(1, 21) = 4.87, *p*<.05. A trend towards significance for the white/bad (*M* = 2; *SD* = 5.9 fixation count per AOI) and black/good (*M* = 9.1 *SD* = 15.7 fixation count per AOI) pairs of the initial blocks *F*(1, 21) = 3.58, *p* = .06 was found.

Fixation duration was higher for black/good (*M* = 4005.5; *SD* = 7687.7 ms) than white/bad (*M* = 605.9; *SD* = 2419.3 ms); *F*(1, 24) = 4.45, *p*<.05 in the good left condition reversed blocks, and for black/good (*M* = 3344.6; *SD* = 6780.7 ms) than white/bad (*M* = 943.5; *SD* = 2938.4 ms); *F*(1, 21) = 4.84, *p*<.05 in the good right condition initial blocks. No significant effect of duration was found for white/good (*M* = 3641.1; *SD* = 7191 ms) and black/bad (*M* = 1179.6; *SD* = 3792.4 ms) in the initial blocks of the good left condition, *F*(1, 24) = 2.29, *p*>.05) and for black/bad (*M* = 2974.7; *SD* = 3711.3 ms) and white/good (*M* = 1090.6; *SD* = 1536.2 ms) in the reversed blocks of the good right condition, *F*(1, 21) = 2.32, *p*>.05).

A repeated-measure ANOVA between subjects was done. No difference between the experimental groups was found in the control blocks for both number and duration fixation. In the initial blocks a significant effect of position was found between groups for number of fixation *F*(1, 21) = 8.584, *p* = .000. Subjects tended to gaze for more times towards the white/good pair indifferently from its position. Moreover, in the initial blocks a significant interaction between group and position was found for fixation duration *F*(1, 21) = 5.244, *p* = .032. In the reversed blocks a significant effect of position was found between groups for both fixation number *F*(1, 21) = 15.40, *p* = .001, and duration *F*(1, 21) = 9.142, *p* = .006.

Multiple linear regression analysis revealed that fixation number and duration were significant predictors of IAT scores *R^2^* = .106, *F*(2, 44) = 2.62, *p*>.05, fixation number *β* = .80, *p*<.05; fixation duration *β* = -.78, *p*<.05). A significant correlation between fixation number and duration was found for both good left condition (initial blocks, r = 0.87, p<.05; reversed blocks, r = 0.95, p<.05) and good right condition (initial blocks, r = 0.86, p <.05; reversed blocks, r = 0.89, p<.05).

### Discussion

Study 1b was designed to exclude or explain any influence of lateralization on eye movements. A lateralization effect was found demonstrating that target position affected fixation number independently of the IAT. However, when the unexpected category/attribute pair, i.e., white/bad was presented on the left and black/good on the right, fixation number for white/bad decreased while on for black/good increased. Results demonstrate that category/attribute pairings that disconfirm our implicit out-group prejudices (i.e., black/good) are more salient than pairings that confirm out-group prejudices (i.e., black/bad) or confirm/disconfirm in-group automatic preferences (i.e., white/good and white/bad) [20], [21]. Study 1b, therefore, revealed that when lateralization is taken into account only fixation duration is predictive of implicit attitudes.

## General Discussion and Conclusions

This study investigated whether eye movement fixation number and/or duration could predict implicit attitudes as measured by the IAT. Embodied cognition theories can be utilized to help explain the relationship between implicit attitudes as measured by the IAT and fixation duration. For embodied cognition, eye movements hold a central role in social cognitive processes via mechanisms that situate conceptualizations [36]. During the IAT, participants' attention – in terms of fixation duration – was focused on the pair that disconfirmed their implicit negative preference, i.e., black/good. These findings are in line with embodied cognition theories in that eye movements should increase in number and/or duration when psychological attributes are incompatible with cognitive processes [11], [37]. Eye movements can work in concert with belief systems that underlie implicit attitudes [14], [22], [23].

Fixation duration has been found to positively correlate with task difficulty level [38], thus providing a valid measure to identify when attentional processing or cognitive load increases. These findings are also confirmed by recent studies showing that the difficulty in visual and cognitive processing of the scene modulates fixation durations [39]–[41] and microsaccades [33]. Considering the present research, higher attentional processing or cognitive load increases would occur when category/attribute pairs mismatch participant implicit prejudices. Other bio-behavioral measures, such as pupil dilation, Heart Rate (HR) or Galvanic Skin Response (GSR) have also been found to be valid measures for identifying increased processing demands [42] and future research should therefore take into account the relationship between these other physiological measures and the IAT to better understand the relationship between arousal and cognitive load during the IAT, especially when category/attribute pairs mismatch participant implicit prejudices.

Because most people read from the upper left to the lower right [34], Study 1b systematically investigated the effect of lateralization on visual information processing. Results demonstrated a strong relationship between fixation number and lateralization leaving only fixation duration as a valid predictor of implicit attitudes. Consistent with previous salience research [20], lateralization was constrained by the strong saliency of the black/good pair that mismatched one's implicit prejudice.

Several other questions remain. These include, for example, whether the relationship between eye movements is consistent across other versions of the IAT, whether eye-tracking can be useful to gain insight into how we represent other social cognition concepts, and whether fixation number and not fixation duration is consistently influenced by the lateral presentation of those concepts. Given the importance of implicit representations in our understanding and evaluation of others and ourselves we see the use of eye-tracking methodologies as a useful tool to explore these issues.
